# Pulsed actomyosin contractions in morphogenesis

**DOI:** 10.12688/f1000research.20874.1

**Published:** 2020-02-25

**Authors:** Ann Sutherland, Alyssa Lesko

**Affiliations:** 1 Department of Cell Biology, University of Virginia Health System, Charlottesville, VA, USA

**Keywords:** actomyosin, morphogenesis, apical constriction, pulsed contractions

## Abstract

Cell and tissue shape changes are the fundamental elements of morphogenesis that drive normal development of embryos into fully functional organisms. This requires a variety of cellular processes including establishment and maintenance of polarity, tissue growth and apoptosis, and cell differentiation, rearrangement, and migration. It is widely appreciated that the cytoskeletal networks play an important role in regulating many of these processes and, in particular, that pulsed actomyosin contractions are a core cellular mechanism driving cell shape changes and cell rearrangement. In this review, we discuss the role of pulsed actomyosin contractions during developmental morphogenesis, advances in our understanding of the mechanisms regulating actomyosin pulsing, and novel techniques to probe the role of pulsed actomyosin processes in *in vivo *model systems.

## Introduction

Most embryos are initially relatively spherical and undergo extensive morphogenetic changes to generate the final form of the organism. Morphogenesis occurs through overall changes in tissue shape and organization resulting from coordinated behaviors at the cellular level. A key element of many of these coordinated behaviors is actomyosin contractility, exerting force within the cell as well as across cells through connections at adherens junctions. Pulsed contractions, generated by myosin II motors and the rapid association and disassociation of F-actin and myosin II (reviewed in 1– 3), function to propagate cell shape changes and to generate epithelial cell rearrangement ( 4 and reviewed in 5, illustrated in 3). We will refer to this contractile function as actomyosin pulsing throughout this review.

The past decade has seen a new focus on the dynamics of actomyosin contractility and the importance of pulsed actomyosin contractions for cell and tissue shape changes such as apical constriction, epithelial folding, and tissue extension and closure. Apical constriction is required for the bending and folding of epithelia and occurs during the formation of tubes, cell ingression, and extrusion of apoptotic or delaminating cells (reviewed in 6). Columnar epithelial cells shorten their apical edge in order to become wedge shaped through the process of apical constriction (reviewed in 6). Actomyosin pulsing drives polarization of the *Caenorhabditis elegans* zygote, as well as the ingression of endodermal, mesodermal, and germline precursors from the surface of the embryo to the interior blastocoel space during gastrulation in *C. elegans*. Actomyosin pulsing is also important for tube formation in the *Drosophila* leg, salivary, and renal tissue ^7– 9^, as well as the *Xenopus* neural epithelium ^10^. Additionally, an imbalance of actomyosin contractility in *Drosophila* ventral furrow cells leads to polarized apical constriction along the dorsoventral axis, causing longitudinal folding of the tissue and the internalization of mesodermal precursors during gastrulation ^11– 14^. Actomyosin pulse-driven apical constriction also drives apoptotic extrusion of human colon cancer cells and delamination and ingression of neuroblasts during *Drosophila* gastrulation ^15– 17^.

Our understanding of actomyosin network organization and the processes that drive actomyosin pulsing has been rapidly evolving. We will describe several models that have provided insights into the role of actomyosin pulsing in developmental morphogenesis and discuss recent advances in experimental tools that will help further clarify these mechanisms.

## Models of pulsed actomyosin contraction

Following the initial description of actomyosin pulsing in the *C. elegans* zygote ^18^, studies of *Drosophila* embryogenesis further demonstrated the importance of actomyosin contraction for cell shape changes and morphogenesis. In particular, the processes of gastrulation and dorsal closure have provided new insights into the importance of actomyosin pulsing to tissue morphogenesis. Gastrulation is initiated by apical constriction of a row of cells on the ventral side of the embryo, leading to ventral furrow formation and subsequent invagination and delamination of the presumptive mesodermal cells ^19^. Dorsal closure, on the other hand, is the process of closure of a gap in the dorsal epithelial sheet resulting from germband retraction. The epithelial sheets on either side of the hole are drawn together and fuse at the dorsal midline to cover the underlying amnioserosa ^20^. Live imaging of actin and myosin during dorsal closure showed that myosin II was localized with F-actin in a supracellular purse string at the margins of the converging epidermal cells ^21^. Contraction of this actomyosin cable was found to promote dorsal closure in coordination with apical contraction of the amnioserosa cells, providing a model of coordinated epidermal and amnioserosa contractile forces, dependent on actin interactions with myosin II ^21^.

Studies of *Drosophila* gastrulation subsequently revealed subcellular details of actomyosin pulsing which challenged the actomyosin purse string model. Live imaging showed myosin II localization to the medial apical cortex of ventral furrow cells, and actin arrayed radially towards the adherens junction, in contrast to the circumferential junctional localization of myosin observed in the supracellular purse string during dorsal closure ^12, 21^. Apical constriction of ventral furrow cells was found to be driven by repeated cycles of contraction of this medioapical actomyosin network followed by a pause in which the apical cell shape is stabilized ^12^. Two critical aspects of this process for efficient constriction of the apical domain were found to be the connection between actin and adherens junction proteins ^6, 22^ and continuous turnover of the actin network ^23^. Loss of connection to the adherens junction led to failure of stabilization of the new apical shape, and inhibition of actin turnover led to loss of connection between the actin and adherens junction ^23^. These data established a new model for apical constriction, in which pulsed actomyosin contractions in the medioapical cortex exert force on the adherens junctions to shrink the apical surface centripetally in a ratcheted manner ( 12, reviewed and illustrated in 3).

Subsequent studies provided evidence for a role for actomyosin pulsing during *Drosophila* dorsal closure, also implicating tension-based control and a ratchet mechanism of pulsing ^24– 26^. Intrinsic pulsatile apical constriction of the amnioserosa cells initiates dorsal closure by bringing the adjacent epidermal cells dorsally, followed by the formation of an actin cable within the epidermal cells ^24, 25^. This supracellular cable maintains epidermal displacement, as the actomyosin cable tension increases slowly throughout dorsal closure, suppressing and stabilizing the forces generated by the amnioserosa actomyosin pulsing to further close the purse string ^25^. In this model, the two tissues coordinate to drive dorsal closure, and the actin cable behaves as a ratchet to compress the amnioserosa cells and promote net contraction of the tissue ^25^. However, more recent work, in which myosin II was selectively eliminated from either the amnioserosa or the epidermal tissue, showed that amnioserosa apical constriction could drive dorsal closure autonomously without the actin cable, while the actin cable was important for zippering integrity ^27^. On the other hand, dorsal closure was delayed in embryos lacking an actin cable, suggesting that the purse string ratchet mechanism may be required to initiate dorsal closure ^27^.

The purse string model as developed in the studies of dorsal closure has informed the analysis of tissue fusion in other situations, in particular closure of the neural tube in mammalian embryos. Neural tube closure involves shape changes and rearrangement of the epithelial cells of the neural plate to form neural folds that come together and fuse along the dorsal side of the embryo ^28^. Imaging of mouse embryos at the late stages of neural tube closure demonstrated that a supracellular actin cable colocalizes with cell junctions to form a continuous purse string structure along the dorsal neural folds of the posterior neuropore. Unlike *Drosophila* dorsal closure, where the actin cable is dispensable for apposition of the epidermal folds, the actin cable in the neural folds is important for closure of the posterior neuropore. Laser ablation of the supracellular cable leads to failure of neural tube closure, indicating that it bears tension and acts to stabilize the neural folds as they fuse ^28^. Actomyosin pulsing has not yet been demonstrated in the mammalian neural epithelium; however, the apical constriction leading to elevation of the neural folds suggests that mechanisms similar to those seen in *Drosophila* ventral furrow formation may also promote tissue shape change during mammalian neurulation, warranting further investigation.

## Actomyosin pulsing during convergence and extension of tissues

Further evidence of the importance of medial actomyosin contractility came from studies of epithelial convergent extension during *Drosophila* germband extension. Germband extension begins following gastrulation and consists of extension of the ventral germband around the posterior end of the embryo. The extension of this epithelium is powered by an extrinsic anterior–posterior force provided by invagination of the posterior midgut ^29^ and convergent extension of the germband epithelium ^30, 31^. Convergent extension is the process by which tissues converge along the mediolateral axis and concomitantly extend along the anterior–posterior axis, thereby narrowing and lengthening the tissue (reviewed in 32, 33). In epithelial tissues, convergent extension involves cell rearrangement through polarized changes in apical intercellular junctions, enabled by actomyosin pulsing ^32^. During germband extension, ectodermal cells of the embryo rearrange through concerted changes in intercellular junctions between groups of four ^30^ or more cells ^31^ (reviewed in 34). Junctions oriented along the dorsoventral axis (vertical junctions) shrink to generate a singular point of contact between all of the cells, followed by expansion of new junctions along the anterior–posterior (horizontal) axis. Initial studies documented polarized junctional localization of myosin II and the requirement for myosin function in junctional dynamics ^30, 31^, while subsequent studies showed that junction shrinkage depended on differential cortical tension ^35^ and pulses of actomyosin contractility ^36^. They identified two distinct pools of myosin II: a medial apical population similar to that seen in the ventral furrow cells and a junctional population ^12, 36– 38^. The vertical junctions go through repeated cycles of shrinking and pausing, where shrinkage is mediated by the medial myosin II pool while the stabilizing pause that follows is regulated by junctional myosin II ^36^. Interestingly, the polarized effect of the medial actomyosin network on the vertical junctions in this system is due to polarized anchorage to adhesion proteins in the horizontal junctions, causing flow of the medial actomyosin pulses toward the vertical junctions ^36^.

Further studies on *Drosophila* germband extension in recent years have elaborated on the role of pulsing in junction remodeling. A recent study has implicated radially directed force in driving tricellular vertex sliding to promote junctional shrinkage, which additionally involves a third pool of myosin at the cell vertices ^39^. Inhibition of myosin II was found to block the extension of new horizontal junctions, implicating medial myosin II as a necessary driver of not only junction shrinkage but also the subsequent growth of the new junctions ^40, 41^. These observations provide an answer to one of the enduring mysteries of the process of epithelial cell rearrangement, namely how the direction of the new junction is determined and how it is elongated. Although medial myosin, and not junctional myosin, is necessary for junctional remodeling, actomyosin pulsing is dependent on anchorage to junctional and apical polarity proteins ^26, 36, 39^. In particular, Canoe, the *Drosophila* homolog of afadin which links junctional and cytoskeletal proteins, has been found to provide the necessary connections to E-cadherin for force transduction ^37^, and a very recent study shows that Polychaetoid, the homologue of the tight junction protein ZO-1, is concomitantly required to maintain adhesion integrity, allowing efficient cell rearrangement ^15, 36, 37, 40, 42^. Furthermore, the duration of pulsed actomyosin contractions in amnioserosa cells during *Drosophila* dorsal closure is regulated by Bazooka, the *Drosophila* homolog of the apical protein Par3 ^26^.

What is not clear from these data is the nature of the ratchet, i.e. how the contractile changes are stabilized between contractile pulses. While viscoelastic properties of the cell cortex promoting dissipation of the contractile force and actin turnover are clearly significant ^43^, recent studies have revealed an important role for Rab35, a GTPase involved in endosome recycling to the plasma membrane, in mediating membrane dynamics ^44, 45^. Rab35 function is required for internalization of plasma membrane during junctional shrinkage and for establishing a focal point for endocytic pathways ^45^. The internalization of plasma membrane provides the ratchet function to the process; without Rab35, the shrinkage gained by pulsed apical actomyosin contractions reverses during the phase in which the cytoskeleton is dissociated and reforms in preparation for the next contractile phase ^45^. Furthermore, the distribution of Rab35 compartments mirrors the asymmetry (or lack thereof) of junctional shrinkage ^45^. The activity of the small GTPase Rab35 is dependent on activation by its guanine nucleotide exchange factor (GEF), Sbf ^44^; however, loss of Sbf has the additional effect of disrupting the localization of myosin II as well as the balance of contractile behaviors between cells ^44^. Thus, Sbf/Rab35 may act to coordinate actomyosin with membrane trafficking to promote efficient cell shape change.

Actomyosin contractility has also been recently demonstrated to mediate *Xenopus* neural tube extension and *C. elegans* epidermal elongation ^46– 48^. Actomyosin activity reduces *Xenopus* neural tissue stiffness in order to promote elongation of the tissue ^46^. Additionally, accumulations of actomyosin were observed at the shrinking junction of intercalating *Xenopus* neural epithelial cells, consistent with the junction rearrangement model proposed by Rauzi *et al.* as discussed above ^47^. Furthermore, recent work in *C. elegans* body-axis elongation supports a ratchet model where the formin FHOD-1, important for actin capping, bundling, and nucleation, stabilizes actin after remodeling and severing caused by contraction to promote elongation of the tissue ^48^.

Pulsed actomyosin contractions have also been implicated in the extension of mammalian tissues. Elongation of cell–cell contacts during compaction in the eight-cell mouse embryo was found to rely on pulsed actomyosin contraction ^49^. Furthermore, mutations in *Shroom*, an actin regulator, disrupted actomyosin localization, disturbed cell junctions, and caused failure of neural tube closure ^50^, suggesting that the actomyosin network may play a role in apical constriction and convergent extension during mammalian neural tube closure. Further studies are needed to evaluate if actomyosin pulsing promotes proper neural tube development in mammals.

Actomyosin pulsing during morphogenesis has been predominantly studied in epithelial tissue. However, recent evidence suggests that pulsed contractions play a role in morphogenesis of mesenchymal tissues as well. During *Xenopus* gastrulation, actomyosin contraction in the mesoderm causes the shrinkage of anterior and posterior cell junctions, allowing for mediolateral cell intercalation and body-axis extension ( 51 and reviewed in 52). This ratchet mechanism of actomyosin pulsing was also demonstrated in the mesoderm during *Xenopus* neural extension, where the mesenchymal deep neural cells extend lamellipodia mediolaterally and then actomyosin contractility drives the intercalation of these cells with their neighbors ^53– 55^. Together these data provide evidence for a role for actomyosin pulsing in mesoderm morphogenesis. However, the differences between actomyosin organization and contractility in the mesoderm versus epithelial tissues are still not fully understood, given the differences in cellular organization and apparent lack of a medial apical pool of myosin. Interestingly, basolateral intercalation of epithelial cells during *Drosophila* germband extension is driven by active migration and basolateral protrusions similar to mechanisms functioning in *Xenopus* mesoderm extension ^56^. Further investigation is necessary to elucidate the mechanisms influencing actomyosin contraction in the mesoderm and how pulsing may be coordinated between epithelial and mesenchymal cells to regulate morphogenesis.

## Molecular mechanisms of actomyosin pulsing

It is clear that actomyosin pulsing affects morphogenesis and that the organization of actin and myosin II is necessary for pulsing, so the next question is how actomyosin organization is regulated during tissue morphogenesis. A key signaling pathway in the context of actomyosin contractility is the Rho family of small GTPases, comprising Rho GEFs and GTPase-activating proteins (GAPs) that regulate the Rho, Rac, and Cdc42 GTPases. Rho signaling was first identified to affect cell shape changes and early development in *Drosophila* embryos where loss of Rho GEF2 led to disruptions in apical constriction needed to complete gastrulation ^57, 58^. RhoA regulates actin pulsing by controlling actomyosin assembly and disassembly independent of myosin II activation in the early *C. elegans* embryo ^59^. However, myosin-independent actions of RhoA are less common than those that directly involve regulation of myosin. During *C. elegans* ovulation and fertilization, for example, Rho1 activity facilitates myosin-dependent contractions of the spermatheca, the organ which houses sperm and where fertilization occurs, leading to the expulsion of the fertilized egg into the uterus ^60, 61^. Additionally, RhoA functions to regulate myosin II in *Drosophila*, as the Cumberland GAP (C-GAP), a RhoA GAP, influences apical constriction of ventral furrow cells by promoting the medial localization of myosin II in coordination with a RhoA GEF in a cyclical manner to initiate pulsing behavior ^62^. Furthermore, Rho kinase and Rho1 GTP exhibit pulsatile localization to the medial actomyosin network, tuning the network dynamics to promote pulsing contractions during *Drosophila* germband extension ^63^. Recent experiments in *Drosophila* ectoderm further implicate the Rho pathway in actomyosin pulsing and, interestingly, demonstrated that the medial and junctional pools of actomyosin are regulated by two distinct Rho GEFs, Rho GEF2 and Dp114RhoGEF, respectively ^64^. The mediation of actomyosin pulsing through two distinct Rho-mediated mechanisms is further supported by the finding that the Rho G-protein-coupled receptor Gα _12/13_ affects medial actomyosin specifically, with no effect on junctional actomyosin ^65^. RhoA has also been identified as an actin pulse regulator in *Xenopus* junction organization and cytokinesis ^66, 67^.

Although the zippering closure of the mouse neural epithelium has been identified to be reliant on an actin cable ^28^, inhibiting actomyosin cross-linking, F-actin assembly, or myosin II activity does not disrupt neural tube closure ^68^. However, inhibiting Rho kinase or blocking F-actin disassembly prevents closure ^68^. Further studies in mouse whole embryo cultures provided evidence that inhibition of Rho kinase prevented neural tube closure by disrupting apical constriction and actomyosin cable organization ^69^. The results of these studies demonstrate that Rho has a role in regulating neural tube closure and actomyosin organization. However, further studies are needed in order to understand whether actomyosin pulsing is occurring in mammalian systems and, if so, to elucidate the specific mechanisms by which Rho is mediating pulsing and how they compare to those elucidated in *Xenopus* and *Drosophila*.

## Novel techniques to investigate actomyosin pulsing

Although we have made great strides in understanding the role of actomyosin pulsing during morphogenesis, there is still much that is unknown about the cellular and molecular mechanisms driving this contractility. The classic experimental tools to study actin have several limitations which restrict our ability to visualize actin interactions and dynamics, measure forces, and manipulate mechanical and molecular variables *in vivo* ^3^ *.* Importantly, although actomyosin pulsing has been observed in single-cell embryos and *in vitro* cell culture assays ^70– 72^, and actin contraction has been shown in purified actin ^73^, contractile pulsing has not been observed in biochemical assays which lack the cytoskeletal turnover and signaling dynamics observed during actomyosin pulsing *in vivo*. This emphasizes the need for novel tools to visualize pulsing *in vivo*; ** therefore, we will highlight a few techniques that could address the limitations of classical actomyosin contractility experiments.

Single-walled carbon nanotubules can improve the visualization of actomyosin dynamics *in vivo* owing to the fact that they are fluorescent at near-infrared wavelengths and can be used as probes for specific proteins through targeting by short oligonucleotides ^74^. These probes were used in *Xenopus* to investigate the effect of crosslinking on cytoskeletal steady states ^75^. Since they are photostable and minimally disruptive to endogenous tissue and protein, they can be utilized for long periods of time, allowing for the analysis of prolonged actin pulses in addition to analysis of rapid assembly and disassembly during contraction ^75^. With these advantages, carbon nanotubes could be useful for analyzing prolonged actomyosin pulsing during development *in vivo* in order to better understand the dynamics of actomyosin contraction.

The ability to measure tension and force *in vivo* would allow for the investigation of how actomyosin pulsing controls cell shape changes and intercalation during morphogenesis. Optical tweezers can be used to measure tension at cell–cell interfaces by directly manipulating the tissue with a laser and then measuring the deflection of the junction ^76^. The combination of optical tweezers and light-sheet microscopy was utilized to measure the dynamics of tension between cells in the *Drosophila* germband during morphogenesis ^76^. The distribution of actomyosin changed from isotropic to anisotropic throughout extension, consistent with the ratcheted actomyosin contraction model ^76^. Furthermore, inhibition of Rho kinase decreased tension at cell–cell contacts ^76^. More recently, a novel fluorescent probe was developed to measure membrane tension without the need to disturb the cell structures. Fluorescent LIPid Tension Reporter (FliptR) contains a negatively charged carboxylate which allows for the insertion of the probe into the membrane ^77^. In a non-confined space, the two large dithienothiophene (DTT) flippers of FliptR can lay flat, whereas, if pressure is applied, the flippers will twist to become planarized, subsequently changing the fluorescence lifetime ^77^. Using fluorescence lifetime imaging microscopy, the changes in fluorescence lifetime can be quantified and extrapolated as a measure of changes in membrane tension ^77^. These methods could provide insight into the mechanical and molecular mechanisms underlying the ratcheted actomyosin pulsing model by measuring tension and stress forces present at the cell junction, cell membrane, and cytoskeletal network interface with or without chemical inhibition in whole tissues *in vivo*.

Finally, the ability to perturb specific mechanical and molecular properties would allow for a detailed dissection of the mechanisms underlying actomyosin pulsing. One such way to manipulate tissue morphogenesis in a living embryo is through liposomal magnetic nanoparticles ^78^. Magnetic nanoparticles are encapsulated into liposomes and injected into tissue, and an external magnetic field can be applied to the magnets to produce a pulsed force in the tissue ^78^. Apical constriction and subsequently mesoderm invagination were induced by apical pulsing of magnetic particles in *snail* mutant *Drosophila* embryos which lack actomyosin contractility and otherwise present with disrupted morphogenesis ^78^. Moreover, magnetic pulsing stabilized Rho kinase and myosin II, demonstrating that this technique is able to mimic endogenous actomyosin pulsing ^78^. The use of these nanoparticles could identify novel regulators of actomyosin pulsing by inducing ratcheted contractions in mutants with developmental defects. Together, these novel experimental techniques provide a toolset that will allow us to overcome the limitations of current probes and will be valuable in gaining a better understanding of actomyosin pulsing during morphogenesis.

## Conclusion

Actomyosin pulsing has been identified as a necessary regulator of proper morphogenesis in several developmental processes in both invertebrates and vertebrates. Although the molecular mechanisms are not fully understood, pulsed actomyosin contractions require proper organization of the actin cytoskeletal network, formation of active myosin II minifilaments, and connection to cell junctions. Furthermore, evidence from a variety of model systems suggests that Rho plays an important role in regulating this cytoskeletal organization and persistent contractile actin function. The discovery of novel probes and analytical tools has improved upon existing techniques in order to better visualize, measure, and manipulate actin dynamics and forces *in vivo*. The use of these new experimental approaches will promote further examination of the role of actin pulsing in morphogenesis and will allow us to gain critical insight into the mechanisms underlying these mechanical forces.
